# Peer-country support for HIV/AIDS, tuberculosis and malaria programmes, Benin, Burkina Faso, Niger and Togo

**DOI:** 10.2471/BLT.25.294262

**Published:** 2026-09-01

**Authors:** Casimir Manzengo, Mariama Baissa, Laurent Moyenga, Yeba L Kaaga, Raoul Saizonou, Dismas Baza, Ndoungou S Ba, Rose Diasso-Tingueri, Fatima Aboubakar, Winiga L Koudema, Moussa Bachabi, Dissou Affolabi, Codjo Dandonougbo, Sidzabda CB Kompaore, Dieudonné N Soma, Adjima Combary, Abou Yahaya, Oum R Ganda, Alphazazi Soumana, Anoumou C Dagnra, Abdou G Gbadamassi, Abraham Atekpe, Benita Manzengo, Issa Guire, Omarou Seybou Moussa

**Affiliations:** a WHO Regional Office for Africa, Burkina Faso Country Office, 158 Avenue de l'Indépendance, BP 7019 Ouagadougou 03, Burkina Faso.; b World Health Organization Country Office, Niamey, Niger.; c World Health Organization Country Office, Lomé, Togo.; d World Health Organization Country Office, Cotonou, Benin.; e World Health Organization Country Office, Mnazi Mmoja, Zanzibar, United Republic of Tanzania.; f Ministry of Health, Cotonou, Benin.; g Ministry of Health, Ouagadougou, Burkina Faso.; h Ministry of Health, Niamey, Niger.; i Ministry of Health, Lomé, Togo.; j Economic History and International Development, London School of Economics, London, England.

## Abstract

**Problem:**

In the World Health Organization’s Regional Office for Africa, the previous inter-country technical support arrangements could not fully meet the growing and diverse needs of the 47 Member States.

**Approach:**

The Regional Office therefore introduced multicountry assignment teams in 2022 to provide timelier, country-centred and integrated technical assistance. The multicountry assignment team for Benin, Burkina Faso, Niger and Togo organized comparative data analysis, thematic webinars, quarterly data reviews, peer-country visits and a best-practices workshop to strengthen the programme performance in human immunodeficiency virus, tuberculosis, malaria and hepatitis.

**Setting:**

The four West African countries together have about 75 million people and have different programme performance levels. The countries also face different security constraints and service-delivery challenges. These factors create a strong rationale for structured peer learning and adaptation of proven practices.

**Relevant changes:**

The initiative strengthened routine data use for decision-making, promoted peer support between countries and facilities, and contributed to practical service-delivery adaptations, including improved implementation of hepatitis B prevention of mother-to-child transmission after peer learning from Burkina Faso.

**Lessons learnt:**

Structured peer-country support can accelerate programme improvement when it is anchored in routine data, focused on practical bottlenecks and translated into simplified, decentralized and task-shared service-delivery models.

## Résumé

**Problème:**

Au sein du Bureau régional de l’Organisation mondiale de la Santé pour la région Afrique, les anciens mécanismes de soutien technique entre pays ne permettaient pas de répondre pleinement aux besoins croissants et variés des 47 États membres.

**Approche:**

Le Bureau régional a donc mis en place, en 2022, des équipes d’intervention multinationales afin de fournir une assistance technique plus rapide, intégrée et centrée sur les pays. L’équipe d’intervention multinationale chargée du Bénin, du Burkina Faso, du Niger et du Togo a mis en place des analyses comparatives de données, des webinaires thématiques, des examens trimestriels des données, des visites multinationales et un atelier sur les meilleures pratiques afin de renforcer les performances des programmes de lutte contre le virus de l’immunodéficience humaine, la tuberculose, le paludisme et l’hépatite.

**Environnement local:**

Ces quatre pays d’Afrique de l’Ouest totalisent quelque 75 millions d’habitants et présentent des niveaux variables de performance des programmes. Ils sont également confrontés à différentes contraintes de sécurité et à des défis en matière de prestation de services. Ces facteurs justifient pleinement le recours à un apprentissage structuré entre pays et l’adaptation de bonnes pratiques.

**Changements significatifs:**

Cette initiative a renforcé l’utilisation systématique des données pour la prise de décision, favorisé le soutien entre pays et établissements et contribué à des adaptations concrètes de la prestation des services, notamment une meilleure mise en œuvre de la prévention de la transmission mère-enfant de l’hépatite B après un apprentissage mutuel avec le Burkina Faso.

**Leçons tirées:**

Un soutien structuré mutuel entre les pays permet d’accélérer l’amélioration des programmes lorsqu’il s’appuie sur des données systématiques, se concentre sur les obstacles concrets et se traduit par des modèles de prestation de services simplifiés, décentralisés et fondés sur le partage des tâches.

## Resumen

**Situación:**

En la Oficina Regional para África de la Organización Mundial de la Salud, los anteriores mecanismos de apoyo técnico entre países no lograron satisfacer del todo las crecientes y variadas necesidades de los 47 Estados Miembros.

**Enfoque:**

Por este motivo, en el año 2022 la Oficina Regional introdujo los equipos de asignación multinacional para ofrecer apoyo técnico más rápido, integrado y adaptado a cada país. El equipo de asignación multinacional para Benín, Burkina Faso, Níger y Togo organizó análisis de datos comparativos, seminarios web temáticos, revisiones de datos trimestrales, visitas entre países homólogos y un taller de mejores prácticas para reforzar el rendimiento del programa para virus de inmunodeficiencia humana, tuberculosis, malaria y hepatitis.

**Marco regional:**

Los cuatro países de África Occidental tienen aprox. 75 millones de habitantes y muestran diferentes niveles de rendimiento del programa. Estos países también afrontan diversas restricciones de seguridad y retos en la prestación de servicios, factores que constituyen un argumento sólido a favor del aprendizaje estructurado entre países homólogos y la adaptación de prácticas de eficacia probada.

**Cambios importantes:**

La iniciativa consolidó el uso de datos rutinarios para la toma de decisiones, promovió el apoyo mutuo entre países y centros, y facilitó adaptaciones prácticas en la prestación de servicios, incluida una implementación mejorada de la prevención de la transmisión de la hepatitis B de madre a hijo tras el aprendizaje mutuo con Burkina Faso.

**Lecciones aprendidas:**

El apoyo estructurado entre países homólogos puede acelerar la mejora de los programas cuando se basa en datos rutinarios, se centra en los “cuellos de botella” prácticos y se traduce en modelos de prestación de servicios simplificados, descentralizados y de tareas compartidas.

## ملخص

**المشكلة:**

في المكتب الإقليمي لمنظمة الصحة العالمية في أفريقيا، لم تكن ترتيبات الدعم الفني السابقة بين الدول قادرة على تلبية الاحتياجات المتزايدة والمتنوعة للدول الأعضاء البالغ عددها 47 دولة، بشكل كامل.

**الأسلوب:**

قام المكتب الإقليمي بناء على ذلك بتكوين فرق عمل متعددة الدول في عام 2022 لتقديم مساعدة فنية متكاملة وفي الوقت المناسب، مع التركيز على احتياجات كل دولة. وقد نظم فريق العمل متعدد الدول في بنين وبوركينا فاصو والنيجر وتوجو تحليلاً مقارناً للبيانات، وندوات عبر الإنترنت حول مواضيع محددة، ومراجعات ربع سنوية للبيانات، وزيارات ميدانية من الدول النظيرة، وورشة عمل حول أفضل الممارسات لتعزيز أداء البرامج في مجال مكافحة فيروس نقص المناعة البشرية والسل والملاريا والتهاب الكبد.

**المواقع المحلية:**

يبلغ عدد سكان الدول الأربع الواقعة في غرب أفريقيا حوالي 75 مليون نسمة، وتختلف مستويات أداء برامجها. كما تواجه هذه الدول قيوداً أمنية وتحديات مختلفة في تقديم الخدمات. تُشكل هذه العوامل أساسًا قويًا للتعلم التشاركي المنظم وتكييف الممارسات المُتفق عليها.

**التغيّرات ذات الصلة:**

عززت المبادرة استخدام البيانات الروتينية في صنع القرار، وشجعت الدعم المتبادل بين الدول والمرافق الصحية، وساهمت في إدخال تعديلات عملية على تقديم الخدمات، بما في ذلك تحسين تنفيذ برامج الوقاية من انتقال التهاب الكبد الوبائي "ب" من الأم إلى الطفل، وذلك بعد الاستفادة من تجربة بوركينا فاصو.

**الدروس المستفادة:**

يُمكن للدعم المتبادل المنظم بين الدول أن يُسرّع من تحسين البرامج عندما يستند إلى البيانات الروتينية، ويركز على المعوقات العملية، ويُترجم إلى نماذج مبسطة ولا مركزية لتقديم الخدمات، مع توزيع المهام.

## 摘要

**问题:**

在世卫组织非洲区域办事处，原有的国家间技术支持安排不能完全满足 47 个成员国与日俱增且纷繁多元的需求。

**方法:**

因此在 2022 年，该区域办事处引进了多国派驻小组，以提供更加及时、以国家为中心且综合一体的技术协助。贝宁、布基纳法索、尼日尔和多哥的多国派驻小组组织进行了比较数据分析、专题网络研讨会、季度数据评审、国家间同行访问以及最佳实践研讨会，来强化人类免疫缺陷病毒、结核病、疟疾以及肝炎的项目成效。

**当地状况:**

这四个西非国家有大约 7500 万人口，项目成效水平各不相同。同时这些国家还面临着不同的安全方面的制约因素和医疗服务提供挑战。这些因素为结构化同行学习、采纳已验证可行的实践创造了坚实充分的理由。

**相关变化:**

这一举措强化了用于制定决策的常规数据运用，推动了各国和机构之间的同行支持，有助于服务提供做出切合实际的调整，包括向布基纳法索的同行进行学习之后预防乙肝母婴传播的实施也有所改善。

**经验教训:**

当以常规数据为基础，重点关注实际瓶颈，并转化为简单易行、非集中化、任务共担的服务提供模式时，结构化国家间同行支持便可以加快项目改善。

## Резюме

**Проблема:**

В Региональном бюро Всемирной организации здравоохранения для стран Африки прежние механизмы межстрановой технической поддержки не могли в полной мере удовлетворить растущие и разнообразные потребности 47 государств-членов.

**Подход:**

В связи с этим в 2022 году Региональное бюро сформировало многострановые целевые группы для предоставления более оперативной и комплексной технической помощи, ориентированной на потребности стран. Многострановая целевая группа по Бенину, Буркина-Фасо, Нигеру и Того организовала сравнительный анализ данных, тематические вебинары, ежеквартальные обзоры данных, визиты в страны-партнеры и семинар по обмену передовым опытом с целью повышения эффективности программ по борьбе с вирусом иммунодефицита человека, туберкулезом, малярией и гепатитом.

**Местные условия:**

Численность населения этих четырех западноафриканских стран составляет около 75 млн человек, при этом уровни эффективности программ в них различаются. Эти страны также сталкиваются с разными проблемами в сфере безопасности и трудностями при предоставлении услуг. Данные факторы обусловливают острую необходимость в организации структурированного взаимного обучения и адаптации проверенных практик.

**Осуществленные перемены:**

Эта инициатива позволила усилить использование регулярно собираемых данных для принятия решений, способствовала развитию взаимной поддержки между странами и медицинскими учреждениями, а также содействовала практической адаптации моделей предоставления услуг, включая усовершенствование мер по профилактике передачи гепатита B от матери ребенку на основе опыта Буркина-Фасо.

**Выводы:**

Структурированная взаимная поддержка стран-партнеров может ускорить совершенствование программ, если она опирается на регулярно собираемые данные, ориентирована на устранение практических узких мест и воплощается в упрощенных, децентрализованных моделях предоставления услуг с разделением обязанностей.

## Introduction

Previously, the World Health Organization (WHO) Regional Office for Africa provided technical assistance to Member States through three inter-country support teams in Burkina Faso for West Africa, Gabon for Central Africa, and Zimbabwe for Eastern and Southern Africa. However, this approach faced challenges, including ad hoc, vertical and short-term technical assistance that was often delayed, and limited capacity to meet growing demands in a diverse and large region of 47 countries. Additionally, the inter-country teams were located far from most countries they were assigned to support. To address these challenges, in 2022, the Regional Office piloted a new strategy to deliver technical assistance, known as the multicountry assignment teams.^1^^–^^4^

These teams have been established in 11 host countries, each covering three to four countries.^5^ Each team includes professionals in nutrition and food safety, maternal and child health, tropical and vector-borne diseases, human immunodeficiency virus (HIV), tuberculosis and hepatitis, noncommunicable diseases, health financing, laboratory and diagnostics, and service delivery.

Burkina Faso is one of the host countries and supports Benin, Niger and Togo. This paper describes how the Burkina Faso, Benin, Niger and Togo initiative, also known as the BBNT initiative, translated the multicountry assignment team model into practical peer-country support for HIV, tuberculosis and malaria programmes, and related hepatitis interventions.^6^^,^^7^

## Local setting

The countries together have almost 75 million inhabitants. Their Human Development Index values ranged from 0.419 to 0.571 in 2023, which reflects persistent gaps in education, income and access to essential services.^8^ Security challenges, violence and conflict in parts of Burkina Faso and Niger further constrain service delivery and programme performance.

## Approach

Because the countries had different performance profiles and best practices, the initiative began with a comparative analysis of national programme data for HIV, tuberculosis and malaria. The results were used to identify high-performing practices and persistent bottlenecks, after which a series of webinars, quarterly data reviews, peer-country visits and a best-practices workshop were conducted. The workshop brought together about 50 experts from the four countries in Burkina Faso.^9^

### Data analysis

A data analysis in January 2024 identified countries with strong performance in selected areas. Based on this analysis, a webinar was held to present the results to HIV, tuberculosis and malaria programme managers from the countries.

The 2022 HIV data analysed were: number of people living with HIV; percentage of expected people living with HIV who know their status; percentage of people living with HIV who are on antiretroviral therapy (ART); percentage of people living with HIV on ART who achieve viral suppression; and percentage of pregnant woman receiving ART for prevention of mother-to-child transmission.^10^

The tuberculosis data analysed were: tuberculosis incidence (number of cases); percentage of cases notified; percentage tested with rapid diagnostics at time of diagnosis; percentage with known HIV status; percentage of people living with HIV newly enrolled in care who were started on tuberculosis preventive treatment in 2024; and tuberculosis treatment rate for new or relapsed tuberculosis cases in 2023.^11^

For malaria, the data analysed were: estimated number of malaria cases, total confirmed cases, reported deaths, cases tested and treated, and percentage of pregnant women receiving intermittent prophylaxis therapy.^12^

After collecting data from the WHO and Joint United Nations Programme on HIV and AIDS platforms, we analysed time trends for each indicator and compared them with the global targets: 95% for HIV indicators, 90% for tuberculosis and 90% for malaria. The best performances were discussed with the countries to identify the success factors. Through peer review, countries identified concrete examples of strong performance that could inform peer learning. For instance, Benin reached 98% coverage of expected HIV-positive pregnant women needing ART to prevent vertical transmission; Togo initiated treatment for nearly all WHO-estimated tuberculosis cases; Burkina Faso produced weekly malaria monitoring bulletins; and Niger demonstrated a strong community strategy to improve tuberculosis treatment coverage.^9^ These practices provided the operational basis for structured exchange across the countries.

### Capacity-building webinars

Over 10 months, the multicountry assignment team held seven webinars on different topics (Table 1).^9^ The webinars (lasting 2–3 hours) included about 40–60 participants for each integrated session and 20–30 participants for the specific sessions. The participants were programme managers, programme technical staff, monitoring and evaluation officers, partners, WHO staff, and scientists in the countries.

**Table 1:** Webinars conducted under the Benin, Burkina Faso, Niger and Togo initiative for the HIV, tuberculosis and malaria programmes, 2024

Date	Topic	Brief description
**Integrated webinars covering all three programmes (40–60 participants)**		
30 January 2024	Presentation of data analysis	Presentation of the data analysis of the national programmes and partners of the four countries with an emphasis on the good performances in the three programmes
27–29 February 2024	Dissemination of new WHO guidance	Presentation of the new guidelines on HIV, hepatitis and sexually transmitted infections (day 1), on tuberculosis (day 2), and on malaria (day 3). Experts from WHO headquarters and the Regional Office for Africa facilitated these sessions
12 March 2024	Capacity-building on data analysis	The session aimed to strengthen participants’ capacity on the main steps of data analysis in the quarterly review, starting with DHIS2 data extraction through to decision-making
14 October 2024	Integrating noncommunicable diseases	Presentation of the situation related to noncommunicable diseases, especially for people living with HIV and tuberculosis patients, and how to integrate the management of noncommunicable diseases in HIV and tuberculosis clinics
**Specific webinars for each programme (20–30 participants)**		
30 April 2024	Pathway to certification of elimination of mother-to-child transmission	Presentation on the prevention of mother-to-child transmission certification process, starting with the pre-assessment to set up the national validation committee with the four thematic groups recommended by WHO (programme, data, laboratory, and communities and human rights and gender). The session was facilitated by experts from WHO headquarters and the Regional Office for Africa
11 June 2024	Intensified finding of missing tuberculosis cases	Discussions on different strategies to intensify the finding of tuberculosis cases in the countries. The session was facilitated by an expert from the Regional Office for Africa
2 July 2024	Malaria surveillance evaluation	Presentation on the surveillance evaluation tools and the results of the surveillance evaluation conducted in Burkina Faso and Niger, followed by open discussions

HIV: human immunodeficiency virus; WHO: World Health Organization

### Quarterly data reviews

Routine data use is central to the precision public health for Africa agenda, which promotes evidence-informed action to end diseases in Africa. In our initiative, quarterly data reviews followed a practical cycle that linked data extraction, quality assessment, data analysis, decision-making and peer discussion.

The reviews were led by national programme managers, using data extracted from health information software DHIS2 (University of Oslo, Oslo, Norway) by monitoring and evaluation staff, which were analysed collectively by programme teams to support evidence-based decision-making. The findings of the reviews can help identify where a problem is and guide the programme on specific actions to take. By sharing this information between countries, other colleagues can adopt this approach to solve similar problems. Within the initiative, the multicountry assignment team prepared the terms of reference, clarified the review methods and provided catalytic support for preparatory activities. Five reviews were conducted between January 2025 and April 2026. Their findings, particularly subnational analyses of low-performing areas, helped programmes identify where bottlenecks occurred, define targeted corrective actions and share lessons that other countries could adapt for similar challenges. 

### Peer-country visits

Three peer-country visits were conducted to allow in-person problem-solving. Six national experts from Burkina Faso visited Togo (28 July–2 September 2024) to work with Togo’s national HIV, tuberculosis and malaria programmes. This visit was followed by a visit of six experts from Niger to Burkina Faso (2–6 September 2024) and a visit by the Togo team to Benin (9–13 September 2024).

The visits created structured opportunities for hosts and visiting teams to analyse implementation challenges jointly, compare operational solutions and agree on practical recommendations that could be adapted in the field.

### Experience-sharing workshop

The first year of implementation of the initiative concluded with a 4-day experience-sharing workshop in Ouagadougou, Burkina Faso, in October 2024 with 50 participants from the four countries.

After plenary presentations, participants worked in three parallel sessions for HIV, tuberculosis and malaria. Each programme presented practices that had contributed to improved performance, and 14 best practices were discussed across the three disease areas.

The workshop also included field visits to health districts, health facilities and communities. Participants observed how seasonal malaria chemoprevention was combined with destruction of larval breeding sites; how triple elimination services and hepatitis B prevention of mother-to-child transmission were decentralized and task-shared to nurses; and how multidrug-resistant tuberculosis services were decentralized. On the final day, each programme prepared a roadmap for adapting selected best practices to their specific needs and context.

## Relevant changes

The main changes were improved use of data in programme management, effective peer support and more practical implementation of prevention of mother-to-child transmission of hepatitis B.^13^

### Use of data 

Data analysis was the backbone of decision-making and programme prioritization in the countries. The initiative helped to position data use as a shared responsibility of programme teams rather than only monitoring and evaluation staff, with actions increasingly guided by routine data analysis. This approach was inspired by the tuberculosis data-improvement experience at Nkembo Hospital, Gabon.^14^

Subnational disaggregation of key indicators was used to locate performance gaps. In Burkina Faso, for instance, HIV testing for prevention of mother-to-child transmission declined from 88% in the first quarter of 2025 to 82% in the second quarter. The review found that in Banfora health district, only 21 of 42 health facilities provided HIV testing in the second quarter, leading the National AIDS Programme and the Family Health Directorate to organize online supervision to address the gap.

### Effective peer-country support

A situation analysis in early 2024 found that although Togo had strong coverage of prevention of mother-to-child transmission services for HIV and syphilis, coverage of prevention of mother-to-child transmission for hepatitis B lagged because operational guidance was unclear at the health-facility level.

Drawing on Burkina Faso’s simplified prophylaxis-for-all approach and task-sharing with nurses and midwives, WHO facilitated a peer-country visit in June 2024 that included field observation and guideline exchange. After this visit, Togo adopted pragmatic solutions, including targeted hepatitis B birth-dose vaccination and the use of tenofovir disoproxil fumarate-based regimens for prophylaxis during pregnancy. This change contributed to improved performance of the prevention of mother-to-child transmission programme (Fig. 1).

**Fig. 1:**
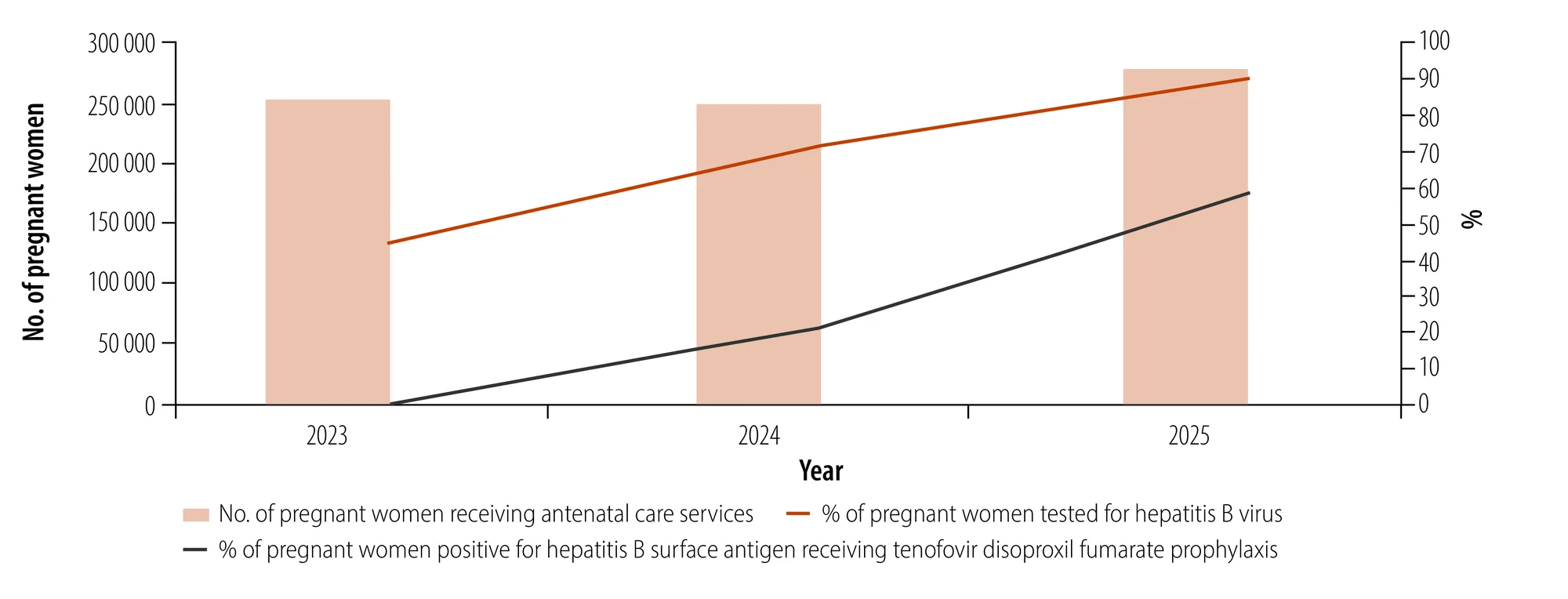
Trend in implementation indicators for prevention of mother-to-child transmission of hepatitis B, Togo, 2023–2025

Togo’s experience of how peer learning, adaptable service-delivery models and WHO technical support can accelerate implementation of prevention of mother-to-child transmission for hepatitis B is documented in the *Global hepatitis report 2026*.^15^

## Lessons learnt

Routine programme activities (e.g. supervision and training) are often planned without sufficient use of implementation data. Data analysis is frequently considered the responsibility of monitoring and evaluation staff, rather than a shared management function. The initiative showed that when programme teams review routine data collectively, they become a practical tool for identifying bottlenecks, prioritizing actions and focusing resources. 

Although countries implement similar HIV, tuberculosis and malaria programmes, field realities and operational constraints differ. Structured exchanges between countries and health facilities help teams compare implementation approaches, identify practical solutions and adapt them to local bottlenecks.

Despite new WHO recommendations on simplified hepatitis B service delivery, some countries still face difficulties in decentralizing prevention of mother-to-child transmission services and implementing task-sharing with nurses and midwives. Technical meetings and guideline exchanges were useful, but the Burkina Faso visit, where these measures were already being implemented, helped countries translate recommendations into practice. 

Previously, programmes relied on parallel data systems, creating challenges of comparability and data quality. Using DHIS2 can make data available across HIV, tuberculosis and malaria programmes, support site-level analysis and provide dashboards to guide decisions. The initiative also showed that some important indicators had not yet been integrated into DHIS2, making their inclusion a priority for strengthening routine data use in the participating countries.

Box 1 summarizes the main lessons learnt from the initiative. Based on this pilot experience, the initiative will be extended to other countries, especially in West Africa, and will maintain the investment in data analysis.

Box 1 **Summary of main lessons learnt**

- Investing in data culture is essential: routine programme data can become a practical management tool when used collectively by programme teams, not only by monitoring and evaluation staff.
- Peer support accelerates programme improvement: structured exchange between countries and health facilities helps identify practical solutions and adapt them to local implementation bottlenecks.
- Simplification, task-shifting and decentralization improve service delivery: pragmatic models, especially for hepatitis B prevention of mother-to-child transmission, can strengthen coverage and make services more accessible in primary health care.

## References

[1] Nastase A, Rajan A, French B, Bhattacharya D. Technical assistance: a practical account of the challenges in design and implementation. Gates Open Res. 2021 Nov 19;4:177. 10.12688/gatesopenres.13205.2 35299599 PMC8920999

[2] Raine L, Ebert C, McDermott J. Exploring technical assistance delivery modalities in obesity prevention. J Nutr Educ Behav. 2018;50:S32.

[3] Scott VC, Jillani Z, Malpert A, Kolodny-Goetz J, Wandersman A. A scoping review of the evaluation and effectiveness of technical assistance. Implement Sci Commun. 2022 Jun 28;3(1):70. 10.1186/s43058-022-00314-1 35765107 PMC9238031

[4] Kanagat N, Chauffour J, Ilunga JF, Yuma Ramazani S, Ovuoraye Ajiwohwodoma JJP, Ibrahim Anas-Kolo S, et al. Country perspectives on improving technical assistance in the health sector. Gates Open Res. 2021 Aug 24;5:141. 10.12688/gatesopenres.13248.1 35224453 PMC8847213

[5] Ba NS, Usman A, Gasasira A, Kabore P, Achu B, Kulausa H, et al.; Ndoungou Salla Ba et al. Transforming technical assistance for more effective health service delivery in Africa: the WHO multi-country assignment teams experience. Front Public Health. 2025 Jun 18;13:1560361. 10.3389/fpubh.2025.1560361 PMC12213535 40606120

[6] Moeti M. Longer and healthier lives for all Africans by 2030: perspectives and action of WHO AFRO. Lancet. 2017 Dec 23;390(10114):2747–9. 10.1016/S0140-6736(17)32128-1 28917962

[7] Promouvoir l'excellence en matière d’assistance technique à travers l’Afrique: évolution des équipes d’affection multipays de l’OMS (MCATS). Brazzaville: World Health Organization Regional Office for Africa; 2025. French.

[8] Data future exchange. List of countries and territories [internet]. New York: United Nations Development Fund; 2023. Available from: https://data.undp.org/countries-and-territories/ [cited 2026 Mar 10].

[9] Initiative BBNT. une approche innovante de collaboration Sud-Sud pour renforcer la lutte contre le VIH/Sida, la tuberculose et le paludisme en Afrique de l’Ouest. Documentation de la première année de mise en œuvre. Ouagadougou: World Health Organization Regional Office for Africa, Burkina Faso Country Office; 2025. French. Available from: https://www.afro.who.int/fr/countries/burkina-faso/publication/initiative-bbnt-documentation-de-la-premiere-annee-de-mise-en-oeuvre [cited 2026 Mar 10].

[10] AIDSinfo. Epidemic & response [internet]. Geneva: UNAIDS; 2024. Available from: https://aidsinfo.unaids.org/ [cited 2026 Mar 10].

[11] Tuberculosis country profiles [internet]. Geneva: World Health Organization; 2025. Available from: https://worldhealthorg.shinyapps.io/tb_profiles/ [cited 2026 Mar 10].

[12] WHO global health observatory. Malaria [internet]. Geneva: World Health Organization; 2025. Available from: https://www.who.int/data/gho/data/themes/malaria [cited 2026 Mar 10].

[13] Première revue trimestrielle des données de lutte contre le VIH/IST/HV, la Tuberculose et le paludisme au Bénin, au Burkina Faso, au Niger et au Togo dans le cadre de l’initiative BBNT. Brazzaville: World Health Organization Regional Office for Africa; 2025. French. Available from: https://www.afro.who.int/fr/publications/premiere-revue-trimestrielle-des-donnees-de-lutte-contre-le-vihisthv-la-tuberculose-et [cited 2026 Mar 10].

[14] Manzengo C, Mavhunga F, Ngatu NR, Lignenguet F, Manguinga S, Nkone GA. TB data improvement in Nkembo health treatment center in Libreville, Gabon. Trop Med Infect Dis. 2026 Mar 27;11(4):90. 10.3390/tropicalmed11040090 42042951 PMC13119969

[15] Global hepatitis report 2026. Geneva: World Health Organization; 2026. Available from: https://iris.who.int/handle/10665/385385 [cited 2026 Aug 13].

